# The Impact of Dyslipidemia on the Health-Related Quality of Life of Korean Females Aged 50 Years and Older

**Published:** 2019-03

**Authors:** Hyejin PARK

**Affiliations:** Department of International Healthcare Management, Daegu Catholic University, Kyungbuk, Republic of Korea

## Dear Editor-in-Chief

Dyslipidemia is one of the major risk factors of cardiovascular disease by contributing to the development and progression of atherosclerosis (1). In Korea, 47.8% of people over 30 yr of age had dyslipidemia in 2015 and the prevalence of dyslipidemia is steadily increasing (2). Particularly, the prevalence of dyslipidemia in women increases significantly after the age of 50 (2). Dyslipidemia has negative effect on health-related quality of life (HRQoL) (3), even though relatively few studies have measured the HRQoL associated with dyslipidemia in women aged 50 yr and over.

To evaluate HRQoL in the target population, many tools, including Medical Outcome Study Short Form-36 (SF-36), EuroQol-5 Dimension (EQ-5D), and Health Utility Index Mark 3 (HUI-3), were developed (4). Among these instruments, EQ-5D is a self-reported questionnaire that consists of five domains of HRQoL and standardized research tools to measure and describe HRQoL (5). Because of high validity and reliability, EQ-5D has recently been widely used in the measurement of HRQoL for patients with disease in many studies. The aim of this study was to investigate the association between dyslipidemia and HRQoL in women over 50 yr of age using the Korea National Health and Nutrition Examination Survey (KNHANES). For this purpose, this study analyzed the relationship between prevalence of dyslipidemia and HRQoL in each of the 5 dimensions of EuroQoL.

This study was undertaken using data from the KNHANES 2014–2015, which included a health and nutrition survey and a medical examination. The KNHANES sample was selected using a stratified multistage cluster sampling design with proportional allocation based on the National Census Registry. From this sample, 3198 women (≥50 yr of age) provided data without values missing for any of the variables analyzed in this study. Participants were asked their demographic characteristics and presence of dyslipidemia. Height and weight were measured with the subjects wearing light clothing and no shoes. BMI was then calculated as weight (kg) divided by the square of height (m^2^).The subjects were categorized as underweight (BMI<18.5), normal (18.5≤BMI<22.9), overweight (23.0 ≤ BMI<24.9), and obese (BMI ≥25.0) according to the WHO definitions for Asian populations (6).

The study protocol was approved by the Korean Ministry of Health and Welfare and was conducted in accordance with the Ethical Principles for Medical Research Involving Human Subjects, as defined by the Helsinki Declaration. The study participants provided written informed consent.

HRQoL was assessed using EQ-5D questionnaire. EQ-5D is a self-reported descriptive system instrument with five health dimensions (mobility, self-care, pain/discomfort, usual activities, and anxiety/depression) each divided into three different levels, namely no problems, some or moderate problems and severe or extreme problems (7). The level scores were used in order to describe an overall measure of perceived HRQoL.

Logistic regression models were used to estimate the odds ratio (OR) and 95% CIs for abnormal (disability) versus normal (no problem) in the categories of EuroQol among participants who reported to have dyslipidemia compared with the reference group (those who reported to not have dyslipidemia). All statistical analyses were conducted using SAS version 9.4 (SAS Institute, Cary, NC, USA).

The mean age and BMI of participants without dyslipidemia were 63.6 and 24.0, respectively. In contrast, the mean age and BMI were 65.6 and 24.4, respectively, in the subjects with dyslipidemia. The EQ-5D index was lower in participants with dyslipidemia (with dyslipidemia: 0.87 ± 0.18, without dyslipidemia: 0.89 ± 0.16, *P*<0.001) suggesting significant impairment of HRQoL in the elderly women with dyslipidemia (Table 1).

**Table 1:** Age, BMI, and EQ-5D of study population by category of dyslipidemia

***Variable***	***All***	***Non-dyslipidemia***	***Dyslipidemia***
N	3198	2430	768
Age (yr)	64.2 ± 9.1	63.6 ± 9.4	65.8 ± 7.8
BMI (kg/m^2^)	24.2 ± 3.3	24.0 ± 3.2	24.9 ± 3.3
EQ-5D	0.89 ± 0.17	0.89 ± 0.16	0.87 ± 0.18
Data are presented as mean ± standard deviation			

Table 2 shows the ORs for disabilities associated with dyslipidemia. The age-adjusted ORs for problems with mobility, self-care, and usual activities were not significantly associated with dyslipidemia. However, compared to those without dyslipidemia, those with dyslipidemia had adjusted ORs for pain/discomfort and anxiety/depression of 1.24 (95% CI: 1.05–1.47, *P*=0.014) and 1.37 (95% CI: 1.12–1.68, *P*=0.002), respectively.

**Table 2:** Age-adjusted odds ratios (95% CI) for disability in the categories of EuroQol by the prevalence of dyslipidemia in Korean women 50 yr of age or older

***EuroQoL domain***	***Non-dylipidemia (n=2430)***	***Dyslipidemia (n=768)***	***P***
EuroQoL-mobility	1.00 (reference)	1.12 (0.93–1.35)	0.229
EuroQoL-self care	1.00 (reference)	1.13 (0.85–1.50)	0.393
EuroQoL-usual activities	1.00 (reference)	1.12 (0.91–1.39)	0.290
EuroQoL-pain/discomfort	1.00 (reference)	1.24 (1.05–1.47)	0.014
EuroQoL-anxiety/depression	1.00 (reference)	1.37 (1.12–1.68)	0.002

Thus, this study showed that dyslipidemia significantly compromised the HRQoL of Korean females aged ≥50 yr. In terms of the five dimensions of the EQ-5D, the ORs for disability in the dimensions of pain/discomfort and anxiety/depression were significantly higher in Korean females with than in those without dyslipidemia. In particular, anxiety/depression exerted the strongest influence on the HRQoL of dyslipidemic subjects, suggesting that the prevention and management of anxiety/depression are important for improving the HRQoL of such individuals.
